# Supplementary material for: High prevalence of Panton-Valentine leukocidin positive, multidrug resistant, Methicillin-resistant Staphylococcus aureus strains circulating among clinical setups in Adamawa and Far North regions of Cameroon
Source: PLoS One. 2022 Jul 8;17(7):e0265118. doi: 10.1371/journal.pone.0265118 (PMC9269376; doi:10.1371/journal.pone.0265118)
Supplement: S1 Table — (DOCX) [file pone.0265118.s001.docx]

**Supplementary Table 1. Oligonucleotide primers used for Sccmec types I to V identification.**

| **Components** | **The volume required for 01 reaction** | **Amplicon size (bp) and specific gene** |
| --- | --- | --- |
| β-F (5’-ATTGCCTTGATAATAGCCYTCT-3’) | 0.4 µL | 937  SCC*mec* II and IV |
| α3-R (5’-TAAAGGCATCAATGCACAAACACT-3’) | 0.4 µL |  |
| 5RmecA-F (5’-TATACCAAACCCGACAACTAC-3’) | 0.2 µL | 359  SCC*mec* V |
| 5R431-R (5’-CGGCTACAGTGATAACATCC-3’) | 0.2 µL |  |
| ccrCF-F (5’-CGTCTATTACAAGATGTTAAGGATAAT-3’) | 0.5 µL | 518  SCC*mec* III and V |
| ccrCR-R (5’-CCTTTATAGACTGGATTATTCAAAATAT-3’) | 0.5 µL |  |
| 1272 F1-F (5’- GCCACTCATAACATATGGAA-3’) | 0.16 µL | 415  SCC*mec* I and IV |
| 1272R1-R (5’- CATCCGAGTGAAACCCAAA-3’) | 0.16 µL |  |
