# Supplementary material for: High prevalence of Panton-Valentine leukocidin positive, multidrug resistant, Methicillin-resistant Staphylococcus aureus strains circulating among clinical setups in Adamawa and Far North regions of Cameroon
Source: PLoS One. 2022 Jul 8;17(7):e0265118. doi: 10.1371/journal.pone.0265118 (PMC9269376; doi:10.1371/journal.pone.0265118)
Supplement: Attachment — Submitted filename: Responses to the reviewers comments-FINAL.docx [file pone.0265118.s004.docx]

**Responses to the reviewers comments**

Dear Sir/Madam thank you for this careful review of our manuscript. We have responded to each question and comment below :

(NB : Line number refers to data from manuscript with track changes)

**Academic Editor**

**2- Please provide an amended statement that declares *all* the funding or sources of support (whether external or internal to your organization) received during this study, as detailed online in our guide for authors at http://journals.plos.org/plosone/s/submit-now.**

- *Financial disclosure statement has been incorporated in the meta data as suggested and in manuscript financial disclosure statement has also been incorporated.*

**2- Please also include the statement “There was no additional external funding received for this study.”**

- *Financial disclosure statement has been incorporated in the meta data as suggested and in manuscript financial disclosure statement has also been incorporated.*

**4- Please include captions for your Supporting Information files at the end of your manuscript, and update any in-text citations to match accordingly.**

- *Thank you for indicating, we have included supporting Information file information at Line 166, 239 and 747.*

**Reviewers 1**

**Title**

**I think the work would benefit from the title that contains the main conclusion of the study (should be derived from the conclusion). Please modify the title.**

- Title has been modified according to suggestion as follows:

*«**High prevalence of Panton-Valentine leukocidin positive, multidrug resistant, Methicillin-resistant Staphylococcus aureus strains circulating among clinical setups in Adamaoua and Far North regions of Cameroon»*

**Abstract:
- The abstract must illustrate the used methods and the most prevalent results (give more hints about methods and results). Besides, rephrase the aim of the work and the main conclusion of your** **findings.**
*More details were included in the abstract highlighting the methods in Line 36 and major results stated in Line 37-58. The conclusion has also been rephrased, Line 59.*

**Introduction (it needs to be more informative). Give a hint about the virulence factors, different infections caused by MRSA, and the mechanism of disease occurrence.**

*Response : Further details have been incorporated in introduction Line 79 to 105*

**The authors should illustrate the public health importance concerning the emergence of multidrug-resistant (MDR) bacterial pathogens that reflect the necessity of new potent and safe antimicrobial agents.**

*Details are included in Line 118-123*

**Rephrase the aim of the work to be clear and better sound.**

**-** *The aim of the study has been rephrased. Lines 123-133*

**Material and methods: Illustrate your methods with subtitles:**

**Add this subtitle: Bacterial Isolation and identification :**
*Subtitle has been added at Line 147*

**Discuss in detail the** **methods of isolation and identification of *S. aureus* and MRSA. Besides, specific references should be added.**

*Details of methods for isolation and identification of S.aureus are provided on line 152 – 165. Moreover suggested references relevant to study are included, reference number 34, 11 and 35.*

**Add the company, city, and country of the used bacterial media and reagents that were used in the biochemical identification of isolates. Also, enumerate all used biochemical reactions.**

*Details of reagents including company and country have been incorporated and given in parenthesis after each Line. Biochemical tests used for preliminary bacterial identification are mentioned in line 155-165.*

**Antimicrobial susceptibility testing**:

**Illustrate the antimicrobial classes of the tested antimicrobial agents within the text**

*Name of each class of antibiotics tested was added and antibiotics were grouped per class*

*Line 175 - 185*

**The authors are advised to classify the tested isolates to MDR , XDR, and PDR as described by Magiorakos *et al.***

*Isolates have been classified as suggested and mentioned in line 323 – 325 and presented in Table 6.*

**Please use PCR to detect antibiotic resistance genes, followed by gene sequencing if possible. Afterward, the correlation between phenotypic and genotypic multidrug resistance should be performed**

*Resistance gene mec A and virulence factors Luk S/F pvl, sccmec were screening by PCR in this study. It is certainly interesting to perform these additional analysis. However, due to funding limitations selected analysis could be performed. Moreover lab closure as a result of COVID 19 positive status of majority individuals (including 2 of our authors) rendered it impossible to perform additional screenings. Therefore this has been listed as a study limitation in discussion Line 470-473.*

**Add more details about the software used in the statistical analyses.**

*Software used in the statistical analyses are mentioned in line 245-247*

**Results:
-Add this subtitle: Phenotypic characteristics of the recovered isolates.**

*The subtitle was added line 254 and more details were provided for the MDR from line 263.* **Illustrate in a new table the occurrence of MDR (Multidrug resistance) among the recovered isolates (illustrate the names of the antimicrobial classes and different antibiotics): No. of strains%Type of resistance, MDR, and XDR Phenotypic multidrug resistance (Antimicrobial classes and different antibiotics).The antibiotic -resistance genes**

*Specific details have been incorporated in new tables 5 and 6 with classes of antibiotics line 322-327 page 12-13*

**Increase the resolution of all figures (it should be 600 dpi)**

*Figure 1 has been reformatted to improve resolution of image.*

**Discussion:
- The authors are advised to illustrate the real impact of their findings without repetition of results.**

*The discussion part has been restructured to avoid repetition of results.*
**-Illustrate the different mechanisms of antimicrobial resistance in *S.aureus*.**

*Appropriate response is provided in line 414-445, 442-449*

**Conclusion
Should be rephrased to be sounded. A real conclusion should focus on the question or claim you articulated in your study, which resolution has been the main objective of your paper?**

*The conclusion has been revised, as suggested Line 476 - 489.*

**RESPONSE TO THE COMMENTS OF REVIEWER 2**

Thank you so much sir/madam for your comments and appreciation to our manuscript. We are going to give responses to your comments in the following paragraphs.
- **The manuscript should be revised for grammar mistakes.**

*Response :* *The manuscript has been thoroughly revised to remove grammatical mistakes.*
- **Please write the scientific names of bacterial pathogens and genes in the correct form all over the manuscript and in the References section (should be italic).**

*Response :* *Well received, correction have been made throughout the manuscript.*
-**The title is broad, please modify the title.**

*Response :* *New title has been proposed.*
**- Add more details about the used methods and most prevalent results in the abstract.**

*Response :* *Details have been included in the abstract.*
-***In the introduction: discuss the public health importance of MRSA and its virulence determinants.***

*Response :* *Details have been included in introduction Line 79 to 105*
-**Improve the aim of work.**

*Response :* *The aim of the study has been rephrased. Lines 123-133*

**Methods:
-Explain the methods of isolation and identification in detail??**

*Response :* *Details of methods for isolation and identification of S.aureus are provided on line 152 – 165.*
-**Specific references should be added to all the used methods and techniques.**

*Response :* *Thank you for pointing this oversight, we have inserted references in relevant sections.*
**- Antimicrobial susceptibility testing: Add the manufacturing company, city, and country for the used reagents and antimicrobial discs.**

*Response :* *We have inserted required information in methods section.*
-**PCR based detection of virulence genes and antimicrobial resistance genes in the most prevalent retrieved bacterial species should be carried out if applicable (or addresses this point in the study limitations)**

*Response :* *Resistance gene mec A and virulence factors Luk S/F pvl, sccmec were screening by PCR in this study. It is certainly interesting to perform these additional analysis. However, due to funding limitations selected analysis could be performed. Moreover lab closure as a result of COVID 19 positive status of majority individuals (including 2 of our authors) rendered it impossible to perform additional screenings. Therefore this has been listed as a study limitation in discussion Line 470-473.*

**Results:
- Discuss in detail the phenotypic characters of the recovered isolates.**

*Response :* *Specific details are provided in the text Line 254-277*
-**Increase the resolution of different Figures: Please improve.**

*Response :* *Figure resolution has been improved.*
-**PCR based detection of virulence genes and antimicrobial resistance genes in the most prevalent retrieved bacterial species should be carried out if applicable (or addresses this point in the study limitations)**

*Response :. This has been listed as a study limitation in discussion Line 470-473.*

**The correlation between the phenotypic and genotypic MDR should be performed.**

*Response :* *Phenotypic data is incorporated in new table (table 6) and details are listed in line 322-355.*

-**Discussion:
- Please improve.**

*Response :* *The discussion section has been revised completely.*
-**Please improve the main conclusion of the manuscript.**

*Response :* *The conclusion has been revised.*
